# LIPCAR levels in plasma-derived extracellular vesicles is associated with left ventricle remodeling post-myocardial infarction

**DOI:** 10.1186/s12967-023-04820-1

**Published:** 2024-01-06

**Authors:** Annie Turkieh, Olivia Beseme, Ouriel Saura, Henri Charrier, Jean-Baptiste Michel, Philippe Amouyel, Thomas Thum, Christophe Bauters, Florence Pinet

**Affiliations:** 1 Inserm, CHU Lille, Institut Pasteur de Lille, U1167- RID-AGE, Université de Lille, Lille, France; 2 U1116-DCAC, Inserm, Université de Lorraine, 54000 Nancy, France; 3 Institute of Molecular and Translational Therapeutic Strategies (IMTTS), Hannover Medical School, Hannover, Germany

**Keywords:** Myocardial infarction, Cardiac remodeling, Extracellular vesicles, LIPCAR, Long noncoding RNA, Biomarker, Plasma

## Abstract

**Background:**

Long Intergenic noncoding RNA predicting CARdiac remodeling (LIPCAR) is a long noncoding RNA identified in plasma of patients after myocardial infarction (MI) to be associated with left ventricle remodeling (LVR). LIPCAR was also shown to be a predictor of early death in heart failure (HF) patients. However, no information regarding the expression of LIPCAR and its function in heart as well as the mechanisms involved in its transport to the circulation is known. The aims of this study are (1) to characterize the transporter of LIPCAR from heart to circulation; (2) to determine whether LIPCAR levels in plasma isolated-extracellular vesicles (EVs) reflect the alteration of its expression in total plasma and could be used as biomarkers of LVR post-MI.

**Methods:**

Since expression of LIPCAR is restricted to human species and the limitation of availability of cardiac biopsy samples, serum-free conditioned culture media from HeLa cells were first used to characterize the extracellular transporter of LIPCAR before validation in EVs isolated from human cardiac biopsies (non-failing and ischemic HF patients) and plasma samples (patients who develop or not LVR post-MI). Differential centrifugation at 20,000*g* and 100,000*g* were performed to isolate the large (lEVs) and small EVs (sEVs), respectively. Western blot and nanoparticle tracking (NTA) analysis were used to characterize the isolated EVs. qRT-PCR analysis was used to quantify LIPCAR in all samples.

**Results:**

We showed that LIPCAR is present in both lEVs and sEVs isolated from all samples. The levels of LIPCAR are higher in lEVs compared to sEVs isolated from HeLa conditioned culture media and cardiac biopsies. No difference of LIPCAR expression was observed in tissue or EVs isolated from cardiac biopsies obtained from ischemic HF patients compared to non-failing patients. Interestingly, LIPCAR levels were increased in lEVs and sEVs isolated from MI patients who develop LVR compared to patients who did not develop LVR.

**Conclusion:**

Our data showed that large EVs are the main extracellular vesicle transporter of LIPCAR from heart into the circulation independently of the status, non-failing or HF, in patients. The levels of LIPCAR in EVs isolated from plasma could be used as biomarkers of LVR in post-MI patients.

**Supplementary Information:**

The online version contains supplementary material available at 10.1186/s12967-023-04820-1.

## Background

Left ventricular remodeling (LVR) following myocardial infarction (MI) is associated with an increased risk of heart failure (HF) and death [1]. In spite of a modern therapeutic approach, LVR remains relatively frequent and difficult to predict in clinical practice, and HF still has a poor prognosis [2–5].

The potential use of long noncoding RNA (LncRNA) as biomarkers for cardiovascular diseases is eliciting an increasing interest [6–8], because they are easily detectable and quantifiable in blood samples, and are more stable than proteins. LncRNAs are noncoding RNAs longer than 200 nucleotides that regulate both gene expression and protein translation [9]. Several lncRNAs were shown to be modulated in the heart or in circulation in different stress conditions such as MI, and shown to be involved in cardiac remodeling by regulating many biological processes including hypertrophy, fibrosis, autophagy and apoptosis [10–20]. LIPCAR (Long Intergenic noncoding RNA predicting CARdiac remodeling) was identified by Kumarswamy et al. as biomarker of cardiac remodeling post-MI [13]. LIPCAR levels were also increased in patients with coronary artery disease (CAD), and more importantly in CAD patients with heart failure compared to those with normal cardiac function [14]. Furthermore, it was shown that increased LIPCAR levels in plasma predict early death of chronic HF patients with reduced ejection fraction [13]. However, the transport of LIPCAR from heart into circulation and the role of this lncRNA in cardiac remodeling and HF post-MI are not yet elucidated.

Noncoding RNAs can be transported to the extracellular environment by binding to proteins or into extracellular vesicles (EVs) [21, 22]. EVs are membrane-bound particles secreted by most cells into the extracellular spaces [23]. They transport RNAs, proteins and lipids and protect them from extracellular degradation playing an important role in intercellular communication. EVs are very heterogeneous: they differ in their content, size and biogenesis; we can distinguish large EVs > 150 nm containing mainly apoptotic bodies and microvesicles of membrane origin, and small EVs < 150 nm containing mainly exosomes of endosomal origin. Interest in studying EVs and their involvement in cardiovascular disease has increased over the past decade [24–27]. Recently, it was shown that EVs-lncRNAs are involved in cardiac remodeling [28–33] and could be used as biomarkers of cardiovascular diseases [34–39]. An interesting study showed that large amount of lncRNAs are modulated in lEVs, sEVs, and cardiomyocytes during hypoxia/reoxygenation [33]. Furthermore, the comparison of lncRNAs profiling of lEVs and sEVs showed that only few lncRNAs are deregulated in both sEVs and lEVs, indicating a selective packaging and sorting mechanism of lncRNAs into specific vesicle subtypes.

The aims of this study are to (1) characterize the transporter of LIPCAR from heart to circulation by studying the expression of this lncRNA in sEVs and lEVs isolated from non-failing and ischemic HF patients; and (2) determine if LIPCAR levels in plasma isolated-EVs reflect the alteration of its expression in total plasma and could be used as biomarkers of LVR post-MI.

## Methods

**Fig. 1:**
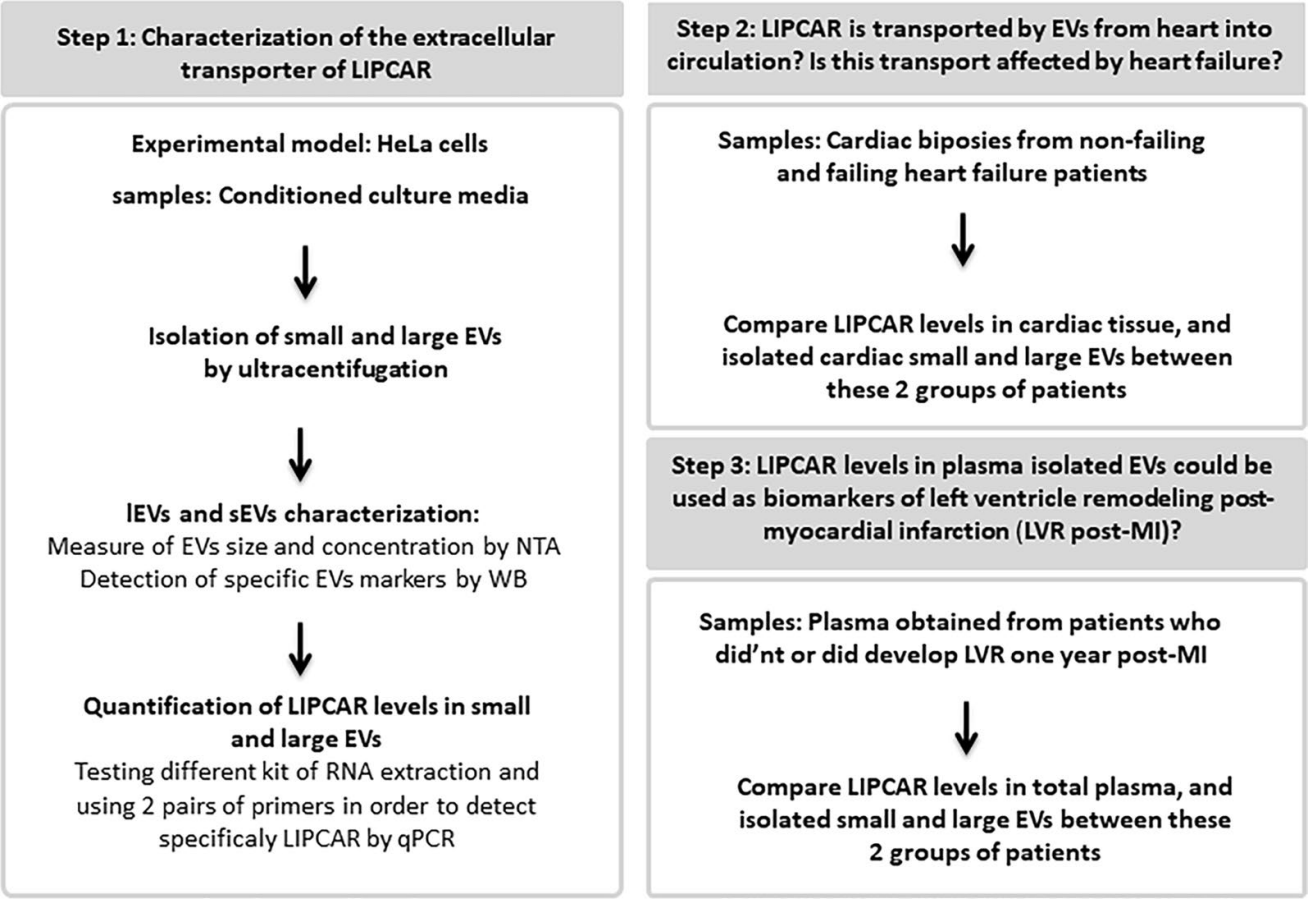
Experimental design of the study. EVs: extracellular vesicles, NTA: nanoparticle tracking analysis, WB: western blot, qPCR: quantitative real time-polymerase chain reaction

The study design is illustrated in Fig. 1.

### Hela cells

HeLa Cells (CCL2, ATCC) were cultured in Dulbecco’s Modified Eagle’s Medium (DMEM Glutamax, 31,966,021, Thermo Fisher Scientific) with addition of 10% (v/v) fetal bovine serum (FBS, Gibco) and 1% penicillin and streptomycin (P/S, 15,140–122, Thermo Fisher Scientific) at 37 °C under 5% CO2 atmosphere. Cells at passage 15 to 20 were used for experiments. Cells were seeded at a density of 1 × 10^6^ cells/petri dish and cultured in complete medium for 48 h. Cells then were serum-deprived for 48 and then counted for normalization. Culture media collected from 4 dishes were pooled (corresponding to one sample for EV isolation) and then stored at − 20 °C.

### Human cardiac samples

Human heart biopsies were obtained from the cardiovascular biobank of Bichat Hospital in Paris (BB-0033–00029, coordinator Dr JB Michel) with approval by the Inserm Institutional Review Board. Patients or their relatives were informed that anonymized tissue will be used for research and given the right to refuse. Eighteen explanted heart tissues were obtained from men patients aged 44 to 77 years who undergoing heart transplantation for end-stage ischemic heart failure (n = 9) or died from non-cardiac causes (n = 9). Samples were quick-frozen and stored at − 80 °C. Note that the samples were thawed several times before using them to isolate EVs.

RNA isolated from human adult cardiomyocytes and cardiac fibroblasts were purchased from Sciencells (# 6335 and # 6215 respectively). Total RNA was prepared from early passage Human cardiac myocytes or fibroblasts -adult using the Qiagen AllPrep DNA/RNA Mini kit.

### Human plasma samples

Plasma samples from REVE-2 (REmodelage VEntriculaire) study are used. This study was approved by the Ethics Committee of the “Centre Hospitalier et Universitaire de Lille” (CP 05/91 of December 13th, 2005) and complies with the Declaration of Helsinski. All patients gave written informed consent. The design of this study is described in detail elsewhere [3]. Briefly, 249 patients with a first anterior wall Q-wave MI were followed for one year by performing serial echographic analysis and collecting serial blood samples.

Plasma samples were collected one year post-MI from patients with (n = 5) or without (n = 5) LVR. Plasma was obtained by centrifugation of blood samples collected in EDTA-containing tubes at 800 g for 10 min, and then stored at − 80 °C. Note that the samples were thawed several times before using them to isolate EVs. The characteristics of the selected patients are summarized in Additional file 1: Table S1.

### Extracellular vesicles (EVs) isolation

EVs were isolated by ultracentrifugation from HeLa conditioned culture media (media from 4 dishes/sample), human plasma (250 µL/sample) and human cardiac biopsies (200 mg/sample were minced and incubated in 6 mL PBS 1X containing 1 mg/mL type II collagenase (LS004174, Worthington) at 37 °C for 30 min with agitation). Conditioned culture media, plasma and cardiac minced tissue-containing PBS were centrifuged at 3000 g to remove debris and dead cells. The supernatants were ultracentrifuged at 20,000*g* (70 min, 4 °C) to pellet large EVs (lEVs), then at 100,000*g* (70 min, 4 °C) to pellet small EVs (sEVs). Rotors used were either Beckman 50.2 Ti or SW-32.1 (337,901/369651 Beckman Coulter France, Villepinte, France).

### Characterization of vesicle number and size by nanoparticle tracking analysis (NTA)

NTAs were performed on EVs samples diluted in PBS with a NanoSight NS300 instrument (Malvern Panalytical) according to the manufacturer’s software manual (NanoSight NS300 User Manual, MAN0541-01-EN-00, 2017). For each sample, several videos of 60 s were recorded and analysed with Nanosight NTA software version 3.2 build 3.1.46. For HeLa cells derived-EVs: 5 videos, camera level 13 and detection threshold 5; for plasma and cardiac derived-EVs: 3 videos, camera level 15 and detection threshold 4.

### EVs protein markers detection by western Blot

Proteins were extracted from HeLa cells derived-lEVs and -sEV in RIPA buffer as previously described [40] and their concentration was measured using Bradford assay (#5,000,006, Bio-Rad Laboratories) according to the manufacturer instructions. Ten µg of proteins were separated on 4–12% SDS-PAGE and transferred on 0.22 µm nitrocellulose membranes (Trans-Blot^®^ Turbo™ Transfert Pack, Bio-rad). After blocking with 5% non-fat dry milk in TBS-Tween 0.1% buffer for 1 h at room temperature, the membranes were incubated with primary antibodies at a dilution 1:1000 in TBS-Tween 0.1% buffer with 5% non-fat dry milk (Tetraspanin CD81: sc-166029, Santa Cruz Biotechnology; MVP (major volt protein): 16,478-1-AP, Proteintech) or with 5% BSA (Tetraspanin CD9: #13,403, Cell Signalling) at 4 °C with gentle shaking overnight. The membranes were then washed three times for 10 min with TBS-Tween 0.1% buffer and incubated for 1 h with the corresponding horseradish peroxidase-labelled secondary antibodies (anti-rabbit IgG NA934V and anti-mouse IgG NA931, GE healthcare) at a dilution 1:5000 in the blocking solution. After three washes with TBS-Tween 0.1% buffer, the membranes were incubated for 5 min with Clarity™ Western ECL Substrate (Bio-Rad Laboratories). Images were acquired using ChemiDoc Imaging System (Bio-Rad Laboratories).

### Quantification of LIPCAR expression by quantitative real time-polymerase chain reaction (qRT-PCR)

RNAs were extracted from cardiac tissue (15 mg) and all EVs samples (from Hela, heart and plasma) with QIAGEN RNeasy Mini Kit, and from total plasma with QIAGEN miRNeasy Serum/Plasma kit as described by the manufacturers’s instructions. TRI Reagent (Ambion) and SeraMir exosome RNA kit (RA808A-1, System Biosciences) were also tested to extract RNA from cardiac tissues and EVs samples respectively as described by the manufacturers’s instructions. For total and EVs-isolated in plasma samples, the synthetic miR-cel 39 was added to verify RNA extraction. RNAs were quantified using NanoVue spectrophotometer and then retrotranscribed using the miScript II RT kit (QIAGEN). Indeed, 50 to 100 ng of RNA (HeLa and cardiac samples) or 12 µL (corresponding to 60% of total RNA isolated from plasma EVs) were mixed with 2 μL of reverse transcriptase enzyme, 2 μL of dNTP mix (10X), 4 μL of HiFlex buffer (5X) and sufficient DNAse/RNAse free water for a total volume of 20 μL. Mixes were then incubated for 1 h at 37 °C on the Biometra Gradient Thermal Cycler followed by 5 min at 95 °C. The cDNAs were then amplified with miScript SYBR Green PCR (QIAGEN) on an Aria Mx Q-PCR system (Agilent Technologies). Indeed, 2.5 µL of 1/40 diluted cDNA was added to 12.5 µL of sybergreen buffer, 2.5 µL of forward and reverse primers (10 µM) and 7.5 µL of RNAase/DNAase free water and amplified according to the following program: Step 1: 95 °C/15 min, Step 2: 94 °C/15 s, Step 3: primer melting temperature/30 s, and Step 4: 70 °C/ 30 s. Steps 2–4 were repeated 40 times. The sequences and the melting temperature (MT) of the different primers (Eurogentec) used were: LIPCAR-1: sense: TAAAGGATGCGTAGGGATGG, antisense: TTCATGATCACGCCCTCATA, MT 60 °C; LIPCAR-2: sense TAATTGTCTGGGTCGCCTGG, antisense: AGGTCAACGATCCCTCCCTT, MT 62 °C; GAPDH: sense: CAGCCTCAAGATCATCAGCA, antisense: TGTGGTCATGAGTCCTTCCA, MT 60 °C; β-actin: sense: GTCCACCGCAAATGCTTCTA, antisense: TGCTGTCACCTTCACCGTTC, MT 60 °C; 18S: sense: CGCCGCTAGAGGTGAAATTC, antisense: TCCGACTTTCGTTCTTGATTA, MT 55 °C. ΔΔCT method was used for data analysis.

### Statistical analysis

Data are expressed as individual value and means ± SEM, and analyzed with GraphPad software version 7.0 (GraphPad, San Diego, CA, USA). Data were compared using non-parametric Mann–Whitney test. The correlation between LIPCAR levels and LVR is determined using the Spearman test. Statistical significance was accepted at the level of *P* < 0.05.

## Results

### Characterization of the extracellular transporter of LIPCAR

Despite its potential value as a biomarker of cardiac remodeling post-MI and heart failure, no information regarding the expression of LIPCAR and its function in heart as well as the mechanisms involved in its transport to the circulation was known.

We first studied whether LIPCAR could be transported by extracellular vesicles (EVs). Given that LIPCAR is only expressed in humans and the cardiac biopsies and plasma samples from patients are limited in quantity, serum free-media from HeLa cells (a model usually used to study and characterize extracellular vesicles [41, 42]) were first used to characterize the extracellular transporter of LIPCAR before validation in EVs isolated from human samples.

**Fig. 2:**
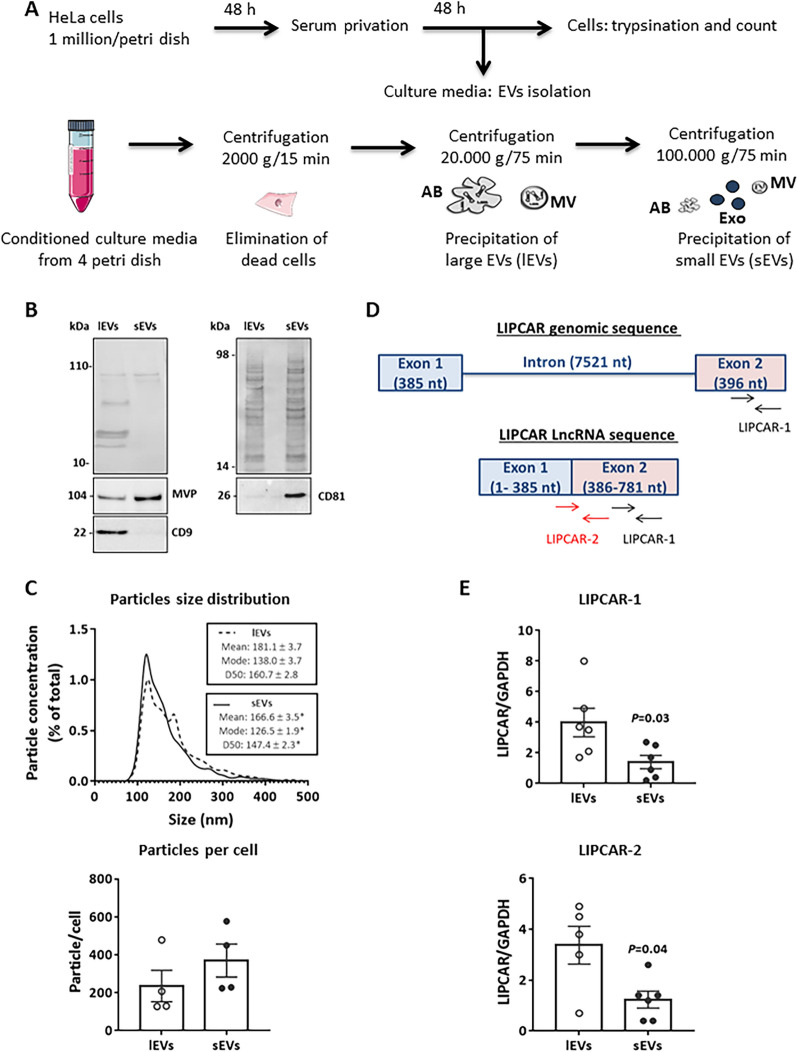
Characterization of HeLa derived-EVs. **A** Protocol of extracellular vesicles (EVs) production and isolation from HeLa cells. AB: apoptotic bodies, MV: microvesicles, Exo: exosomes. **B** Ponceau Red (tops) and western blot of tetraspanins (CD9 and CD81) and major volt protein (MVP) in large (lEVs) and small (sEVs) EVs isolated from HeLa conditioned culture media. **C** Nanoparticle tracking analysis (NTA) of isolated lEVs and sEVs quantifying their size distribution (top) and number of particles produced by cell (bottom). Statistical significance was determined by Wilcoxon-Mann Whitney test *p < 0.05. Data are obtained from 4 independent experiments. **D** Schematic representation of genomic and lncRNA sequences of LIPCAR. The black and red arrows correspond respectively to sequences amplified by LIPCAR-1 and LIPCAR-2 pairs of primers. **E** Quantification by qRT-PCR of LIPCAR levels in large (lEVs) and small (sEVs) extracellular vesicles isolated from HeLa conditioned culture media by using LIPCAR-1 and LIPCAR-2 primers (n = 6/group). GAPDH was used for normalization. Statistical significance was determined by Wilcoxon-Mann Whitney test and significant *P* values are indicated

Differential centrifugation was used to isolate large (lEVs) and small (sEVs) EVs from conditioned culture media (Fig. 2A). Western blots analysis showed a different protein constitution between sEVs and lEVs validating the separation of these two populations of vesicles (Fig. 2B). Indeed, western blots analysis showed increased MVP and CD81 proteins levels in sEVs compared to lEVs, and CD9 is mainly expressed in lEVs (Fig. 2B). NTA analysis also showed a different size distribution between sEVs and lEVs, and a high concentration of both lEVs (1.57 ± 0.8 × 10^10^ particles/mL) and sEVs (2.43 ± 0.88 × 10^10^ particles/mL) with a significant increase of mode, mean and median particle size of lEVs compared to sEVs (Fig. 2C, top). Regarding the amount of particles produced per cell, we observed a non-significant increase in the mean number of sEVs compared to lEVs (Fig. 2C, bottom). Furthermore, the ratio sEVs/lEVs was above 1 in each experiment performed (Additional file 1: Table S2) suggesting a higher production of sEVs compared to lEVs by HeLa cells.

After validation of EVs isolation protocol, the expression of LIPCAR in EVs was then quantified by qRT-PCR. LIPCAR is a mitochondrial long non-coding RNA of 781 nucleotides (nt) whose the 385–781 nt sequence has a strong homology to several nuclear chromosomes sequences. As primers of LIPCAR used in literature, named here LIPCAR-1, are internal to this sequence (528–714 nt, Fig. 2D), and it was shown that cancer derived-EVs could transport DNA [43], we performed several tests to quantify specifically LIPCAR in our conditions. First, we used the seraMiR kit described to extract RNA and quantify other long noncoding RNAs in exosomes [44]. Using the LIPCAR-1 primers, qRT-PCR analysis showed an unspecific amplification of LIPCAR after seraMir extraction (data not shown). Second, we extracted RNA using QIAGEN kit which contain DNA column to eliminate most nuclear contamination and we showed more specificity to quantify RNA from all EV samples. We compared the expression of LIPCAR in lEVs and sEVs, and we showed the presence of both HeLa derived-EVs, and its level is significantly higher in lEVs than sEVs (Fig. 2E left).

Furthermore, it was shown that mitochondria or mitochondrial DNA could be transported by EVs to extracellular compartments [45–48]. In order to detect specifically the lncRNA LIPCAR in EVs and not a fragment of mitochondrial genome, we used another LIPCAR pair of primers, named here LIPCAR-2 which targets the 375–516 sequence that could be only amplified when LIPCAR is transcribed (Fig. 2D). Using these primers, we validated that lncRNA LIPCAR is transported by HeLa derived-EVs and mainly by lEVs than sEVs (Fig. 2E right).

In conclusion, using 2 different pairs of primers, we confirmed a significant higher specific expression of LIPCAR in lEVs.

### *LIPCAR is transported by cardiac extracellular vesicles,* predominantly by lEVs, in non-failing and failing heart patients

**Fig. 3:**
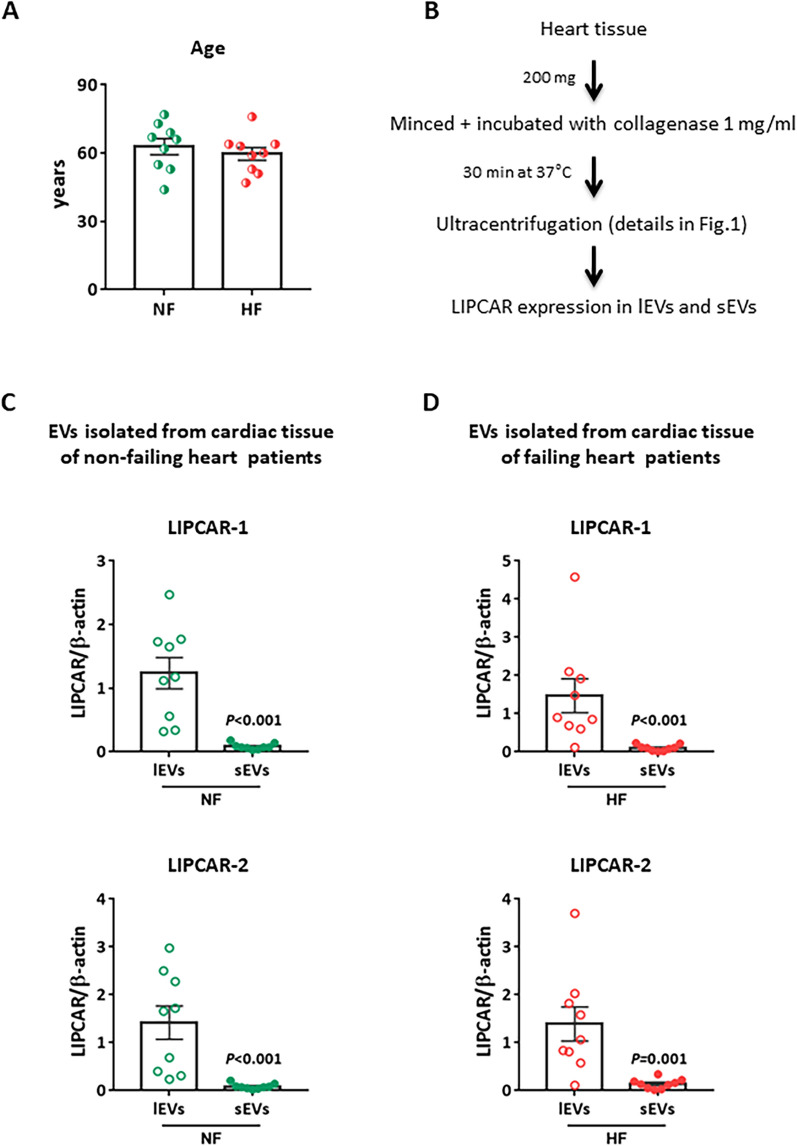
LIPCAR is transported by cardiac EVs, predominantly by lEVs, in non-failing and failing heart patients. **A** Non-failing (NF) and heart failing (HF) patients were age-matched. **B** Isolation procedure of EVs from cardiac tissue. lEVs: large EVs, sEVs: small EVs. **C**, **D** Comparison of LIPCAR expression in lEVs and sEVs isolated from heart of NF (**C**) and HF (**D**) patients using the 2 pairs of primers (n = 9/group). β-actin was used for normalization. Statistical significance was determined by Wilcoxon-Mann Whitney test and significant *P* values are indicated

First, we analyzed LIPCAR expression in adult human heart by using the same RNA QIAGEN extraction method as for EVs. As expected, qRT-PCR analysis showed that LIPCAR is highly expressed in adult heart tissue and in isolated cardiac cells, cardiomyocytes and fibroblasts (Additional file 1: Table S3). We then compared LIPCAR expression in cardiac tissue obtained from ischemic heart failing (HF) patients to non-failing (NF) heart patients matched for age (Fig. 3A) and we found no difference on LIPCAR expression between these 2 groups of patients (Additional file 1: Fig. S1A). We then isolated, by ultracentrifugation, lEVs and sEVs from frozen cardiac tissue obtained from NF and HF patients to determine whether LIPCAR is transported from heart to extracellular compartment by EVs (Fig. 3B). We previously showed by NTA analysis that heart secreted more lEVs than sEVs with a different size distribution profile, and that lEVs and sEVs isolated from ischemic HF patients have the same size and concentration as those isolated from NF heart patients [49]. qRT-PCR analysis using the LIPCAR-1 and LIPCAR-2 pairs of primers showed that LIPCAR is present in cardiac EVs and its level is significantly higher in lEVs than sEVs either in NF (Fig. 3C) and HF (Fig. 3D) patients. However, no difference was observed concerning LIPCAR expression in cardiac lEVs (Additional file 1: Fig. S1B) and sEVs (Additional file 1: Fig. S1C) obtained from HF patients compared to NF heart patients.

In conclusion, these data suggest that LIPCAR is transported to extracellular compartments by cardiac EVs independently of the status, non-failing or failing heart of patients.

### LIPCAR level is increased in EVs-isolated from plasma of LVR post-MI patients compared to non LVR patients

**Fig. 4:**
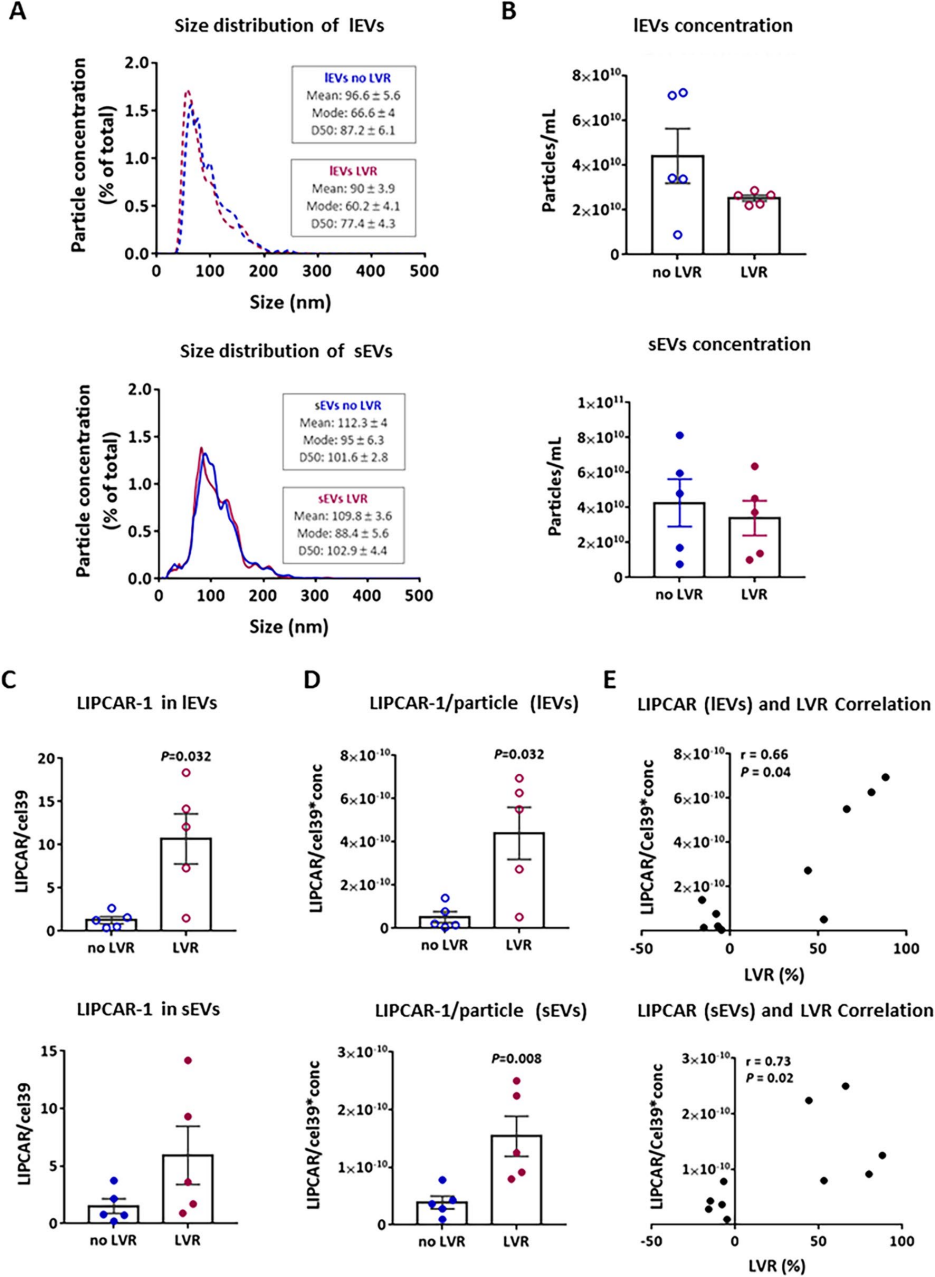
LIPCAR levels are increased in EVs-isolated from plasma of MI patients with left ventricle remodelling (LVR). **A**, **B** Nanoparticle tracking analysis (NTA) of EVs isolated from plasma of MI patients with (LVR) or without LVR (no LVR) (n = 5/group). Size distribution (**A**) and concentration (**B**) of lEVs (top) and sEVs (bottom). **C** Quantification by qRT-PCR of LIPCAR levels in plasma-derived lEVs (top) and sEVs (bottom). The synthetic miR-cel39 was added during RNA extraction and used to normalize LIPCAR levels. **D** Graphs showing the levels of LIPCAR in lEV (top) or sEV (bottom) after normalization by particles concentration of each sample. **E** Correlation between LVR and LIPCAR levels in plasma-derived lEVs (top) and s-EVs (bottom). Statistical significance was determined by Wilcoxon-Mann Whitney test or Spearman test and only significant *P* values are indicated

As previously shown, plasma LIPCAR levels were associated to LVR post-MI in patients from REVE-2 study [13]. We used the same technological approach described above to determine whether LIPCAR is present in plasma derived-EVs and whether its amount in EVs could be used as potential biomarkers of LVR post-MI. We selected 10 patients from REVE-2 study: 5 patients without (non LVR) and 5 who developed LVR (LVR) matched for age (Additional file 1: Fig. S2A and SB). We observed that LIPCAR levels are significantly increased in plasma of LVR patients at one year post-MI compared to non LVR patients (Additional file 1: Fig. S2C), and are positively correlated with LVR (Additional file 1: Fig. S2D). lEVs and sEVs were then isolated from 250 µl of plasma by ultracentrifugation (as shown in Fig. 2A) and used for NTA and qRT-PCR analysis. No difference in size distribution profile and concentration of lEVs and sEVs isolated from LVR patients compared to no LVR patients was observed (Fig. 4A, B). Note that lEVs are very sensitive to freezing/thawing which may explain the small size of the lEVs observed here. qRT-PCR analysis showed that LIPCAR is present in plasma-derived EVs. We showed that LIPCAR levels normalized by miR-cel39 (a synthetic miR used as control for RNA extraction) is only significantly increased in lEVs isolated from LVR patients compared to non LVR patients (Fig. 4C). However, when the concentration of particles isolated per patient has been taken into consideration, LIPCAR levels have been shown to be significantly increased in both type of EVs isolated from LVR patients compared to non LVR patients (Fig. 4D). As observed in total plasma, LIPCAR levels in plasma derived-sEVs and -lEVs are positively correlated with LVR (Fig. 4E). To investigate whether LIPCAR levels in total plasma and plasma-derived EVs are associated with cardiac remodeling rather than cardiac dysfunction, we compared ejection fraction and plasmatic levels of brain natriuretic peptide (BNP) between non LVR and LVR patients. We observed that there is no significant difference between non LVR and LVR patients with all patients having a normal cardiac function with EF > 40% and BNP levels < 100 pg/mL(Additional file 1: Fig. S2E and S2F). These results suggest that the levels of LIPCAR in plasma isolated-EVs reflect the alteration of its expression in total plasma and could be used as biomarkers of LVR post-MI.

## Discussion

EVs are considered as transporters of biomarkers for the diagnosis of cardiac diseases [25–27] and play an important role in cell-to-cell communication during physiological and pathological processes [24, 50]. It was shown that lncRNAs could be transported to extracellular compartment by sEVs and/or lEVs indicating a selective packaging and sorting mechanism of lncRNAs into specific vesicle subtypes [33]. Furthermore, several studies showed that EVs-lncRNAs are involved in cardiac injury and remodeling [28–33] and could be used as biomarkers of cardiovascular diseases [34–38]. Here we showed, for the first time, that LIPCAR is transported by both types of EVs, predominantly by lEVs from heart to circulation and that LIPCAR levels in EVs-derived plasma are associated with cardiac remodeling post-MI. Using two different primers, we confirmed that LIPCAR, and not a fragment of this lncRNA or mitochondria, is transported by EVs suggesting that LIPCAR could be active by playing a role in intercellular communication.

Apart from its potential value as biomarker of cardiovascular disease [13, 14, 51–55], very little data exists on the role of LIPCAR in humans during physiological and pathological conditions. It has been shown that LIPCAR could be involved in atherosclerosis by promoting cell proliferation, migration and phenotypic switch of vascular smooth muscle cells [56]. Furthermore, LIPCAR could contribute to atrial fibrillation by inducing atrial fibrosis via modulating the TGF-β/smad pathway [57]. However, no data exist on the expression of LIPCAR in the heart and its role in cardiac remodeling and HF post-MI.

As LIPCAR is only expressed in humans and its plasmatic levels are associated with HF severity [53], we compared its expression in cardiac biopsies obtained from non-failing patients with those obtained from patients who undergoing heart transplantation for end-stage ischemic heart failure. Since lncRNAs expression could be altered differently in cells and vesicle subtypes [33], we quantified LIPCAR levels in cardiac tissues, and cardiac sEVs and lEVs. No difference of LIPCAR levels in cardiac tissues and EVs was observed between NF and HF patients. However, we showed for the first time that LIPCAR levels were significantly increased in plasma derived- lEVs and sEVs obtained from MI patients with LVR compared to no LVR patients. Several hypotheses could explain this inconsistency in LIPCAR results between plasma and hearts of post-MI patients. The first one is based on the fact that the REVE-2 cohort only included patients with MI (no control patients), and that plasma LIPCAR levels were compared between patients with and without LVR, whereas cardiac LIPCAR levels were compared between HF patients and control patients with no cardiovascular problems. The second hypothesis is based on the fact that LIPCAR expression was measured in global cardiac tissues, however, recent studies showed that LIPCAR plays a role in cellular proliferation [52–54], suggesting that LIPCAR expression could be altered in cardiac fibroblasts, and not in cardiomyocytes. Finally, the presence of LIPCAR in EVs also suggests that this lncRNA could be secreted by non-cardiac cells and then internalized by cardiomyocytes or fibroblasts to contribute to cardiac remodeling. To confirm these hypotheses, it would be interesting to investigate whether LIPCAR expression is differentially altered in cardiomyocytes and cardiac fibroblasts and their derived EVs during cardiac remodeling and HF post-MI to confirm if the increased level of LIPCAR in circulation could be a part of cardiac origin. As LIPCAR is only expressed in humans and its high sequence homology with several DNA sequences prevents the use of in situ hybridization techniques, pluripotent stem cells-derived cardiomyocytes and cardiac fibroblasts treated with different reagent to induce cardiac hypertrophy and fibrosis, or EVs-containing LIPCAR, could be used to study the expression of LIPCAR and its role in cardiac remodeling.

## Limits of the study

Since LIPCAR is only expressed in humans, we cannot quantify LIPCAR expression in fresh cardiac tissue and vesicles isolated from post-MI patients in order to compare to the corresponding plasma samples. Furthermore, we were not able to use cardiac tissue from animal models mimicking MI as we previously done for other non-coding RNAs [58]. Therefore, cardiac LIPCAR expression was only evaluated in frozen cardiac tissue from non-failing patients who died of non-cardiovascular causes and in failing patients who underwent heart transplantation. Here, we used differential ultracentrifugation, the most commonly used method to isolate EVs from biological fluids, cell culture media and, more recently, from cardiac tissue [49, 59]. However, it should be noted that isolated EVs are not pure; depending on the sample type, aggregated proteins, lipoproteins, or other contaminants may also be isolated. Furthermore, we have isolated human EVs from frozen samples (post-mortem or post-operative cardiac biopsies and plasma cohorts).

## Conclusion

We showed for the first time that LIPCAR is transported by EVs, predominantly by lEVs, from the heart into the circulation. This transport is independent of the status of failing or non-failing patients. The levels of LIPCAR in EVs-derived plasma could be used as biomarkers of LVR in post-MI patients.

### Supplementary Information

**Additional file 1: Figure** **S1.** LIPCAR levels in the heart and cardiac EVs obtained from ischemic failing heart (HF) patients compared to non-failing heart (NF) patients. RNAs were extracted from cardiac tissues or EVs of 9 men patients NF and 9 men patients HF, and LIPCAR levels were quantified by qPCR using the 2 pairs of primers (1 in top and 2 in bottum). 18S was used to normalization. **A** Intracardiac LIPCAR expression in HF patients compared to NF patients. **B**, **C** Comparison of LIPCAR expression in lEVs **B** and sEVs **C** isolated from NF and HF patients. **Figure S2.** Characteristics of selected REVE-2 study patients. **A** Patients with (LVR) or without (no LVR) left ventricle remodeling developed one year post-MI were matched for age **(**n = 5/group). **B** Mean percentage of LVR (% LVR) at 1 year post-MI. % LVR was calculated as: (LVR_1year_- LVR_base_)*100/LVR_base_. **C** LIPCAR levels quantified by qRT-PCR in total plasma collected at one year from no LVR and LVR patients using the pair of primers LIPCAR-1. Statistical significance was determined by Wilcoxon-Mann Whitney test and only significant *p* values are indicated. **D** Correlation between LIPCAR levels in total plasma and % LVR. Statistical significance was determined by Spearman test. **E** Ejection fraction (EF) and **F** plasmatic levels of brain natriuretic peptide (BNP) in non LVR and LVR patients. **Table S1.** Characteristics of selected patients from the REVE-2 study. EF: Ejection fraction, EDV: End-diastolic volume, ESV: End-systolic volume, % LVR: Percentage of left ventricle remodeling, CK: creatine kinase, BNP: brain natriuretic peptide. Statistical significance was determined by Wilcoxon-Mann Whitney test. *P < 0.05, **P < 0.01. **Table S2.** Number of large (lEVs) and small (sEVs) EVs isolated from HeLa conditioned culture media. **Table S3.** LIPCAR expression in adult human cardiac tissue and human cardiac cells.

## Data Availability

All data generated or analysed during this study are included in this published article and its Additional files.

## Abbreviations

CAD: Coronary artery disease
EVs: Extracellular vesicles
HF: Heart failure
lEVs: Large extracellular vesicles
LIPCAR: Long intergenic noncoding RNA predicting cardiac remodeling
LncRNA: Long noncoding RNA
LVR: Left ventricle remodeling
MI: Myocardial infarction
MVP: Major volt protein
NTA: Nanoparticle tracking analysis
qRT-PCR: Quantitative real time-polymerase chain reaction
REVE-2 study: REmodelage VEntriculaire study
sEVs: Small extracellular vesicles
TGF-β: Transforming growth factor beta
WB: Western Blot
