# Supplementary material for: LIPCAR levels in plasma-derived extracellular vesicles is associated with left ventricle remodeling post-myocardial infarction
Source: J Transl Med. 2024 Jan 6;22:31. doi: 10.1186/s12967-023-04820-1 (PMC10771704; doi:10.1186/s12967-023-04820-1)
Supplement: Supplementary file 1 — Additional file 1: Figure S1. LIPCAR levels in the heart and cardiac EVs obtained from ischemic failing heart (HF) patients compared to non-failing heart (NF) patients. RNAs were extracted from cardiac tissues or EVs of 9 men patients NF and 9 men patients HF, and LIPCAR levels were quantified by qPCR using the 2 pairs of primers (1 in top and 2 in bottum). 18S was used to normalization. A Intracardiac LIPCAR expression in HF patients compared to NF patients. B, C Comparison of LIPCAR expression in lEVs B and sEVs C isolated from NF and HF patients. Figure S2. Characteristics of selected REVE-2 study patients. A Patients with (LVR) or without (no LVR) left ventricle remodeling developed one year post-MI were matched for age (n = 5/group). B Mean percentage of LVR (% LVR) at 1 year post-MI. % LVR was calculated as: (LVR1year- LVRbase)*100/LVRbase. C LIPCAR levels quantified by qRT-PCR in total plasma collected at one year from no LVR and LVR patients using the pair of primers LIPCAR-1. Statistical significance was determined by Wilcoxon-Mann Whitney test and only significant p values are indicated. D Correlation between LIPCAR levels in total plasma and % LVR. Statistical significance was determined by Spearman test. E Ejection fraction (EF) and F plasmatic levels of brain natriuretic peptide (BNP) in non LVR and LVR patients. Table S1. Characteristics of selected patients from the REVE-2 study. EF: Ejection fraction, EDV: End-diastolic volume, ESV: End-systolic volume, % LVR: Percentage of left ventricle remodeling, CK: creatine kinase, BNP: brain natriuretic peptide. Statistical significance was determined by Wilcoxon-Mann Whitney test. *P < 0.05, **P < 0.01. Table S2. Number of large (lEVs) and small (sEVs) EVs isolated from HeLa conditioned culture media. Table S3. LIPCAR expression in adult human cardiac tissue and human cardiac cells. [file 12967_2023_4820_MOESM1_ESM.docx]

**SUPPLEMENTAL DATA**

**LIPCAR level in plasma-derived extracellular vesicles is associated with left ventricle remodeling post-myocardial infarction**

**Annie Turkieh^1^, Olivia Beseme^1^, Ouriel Saura^1^, Henri Charrier^1^, Jean-Baptiste Michel^2^, Philippe Amouyel^1^, Thomas Thum^3^, Christophe Bauters^1^, Florence Pinet^1^**

^1^Université de Lille, Inserm, CHU Lille, Institut Pasteur de Lille, U1167- RID-AGE, Lille, France

^2^ U1116-DCAC, Université de Lorraine, Inserm, 54000 Nancy, France.

^3^ Institute of Molecular and Translational Therapeutic Strategies (IMTTS), Hannover Medical School, Hannover, Germany

**Corresponding authors**: Dr. Florence Pinet: [florence.pinet@pasteur-lille.fr](mailto:florence.pinet@pasteur-lille.fr) ; Dr. Annie Turkieh : ani.turkieh@pasteur-lille.fr

**Suppl Figure and tables:**

**
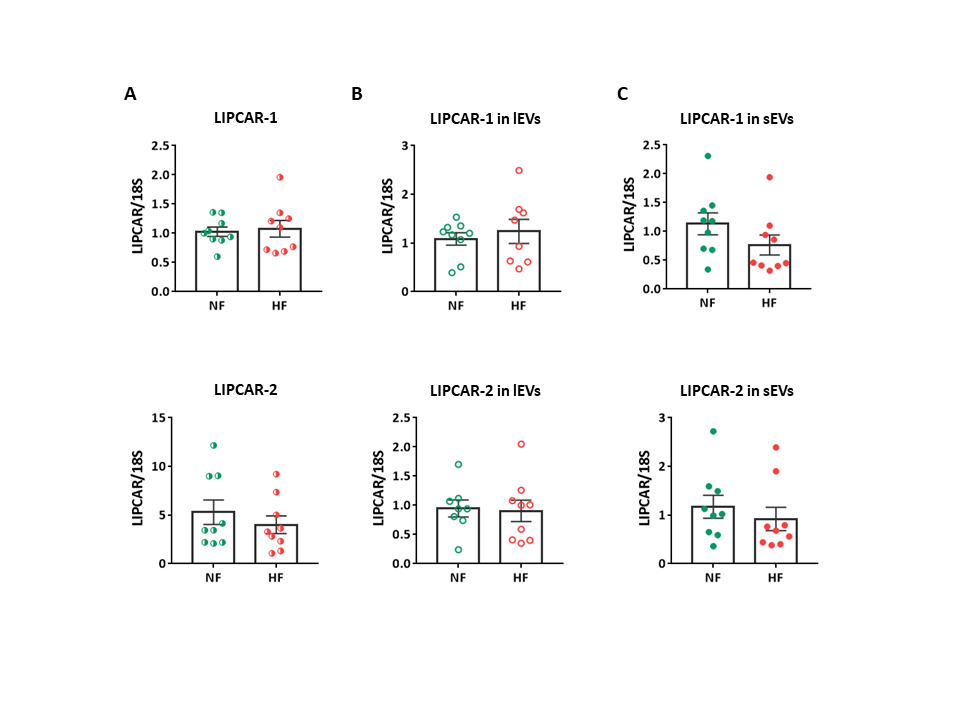
**

**Suppl. Figure 1: LIPCAR levels in the heart and cardiac EVs obtained from ischemic failing heart (HF) patients compared to non-failing heart (NF) patients.** RNAs were extracted from cardiac tissues or EVs of 9 men patients NF and 9 men patients HF, and LIPCAR levels were quantified by qPCR using the 2 pairs of primers (1 in top and 2 in bottum). 18S was used to normalization. **(A)** Intracardiac LIPCAR expression in HF patients compared to NF patients. **(B-C)** Comparison of LIPCAR expression in lEVs **(B)** and sEVs **(C)** isolated from NF and HF patients.

**
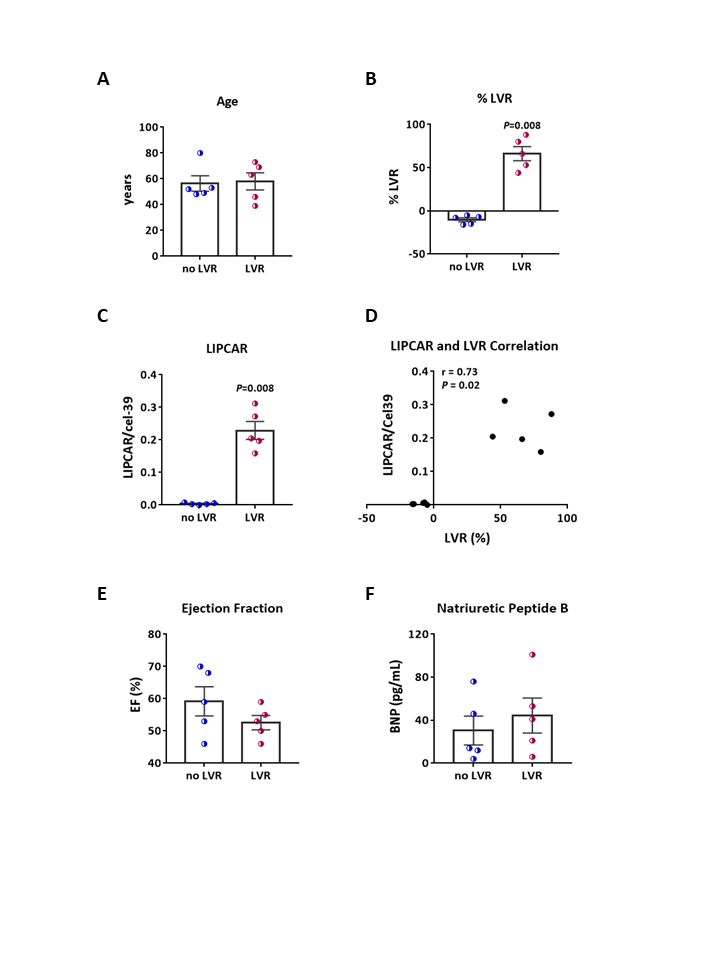
**

**Suppl. Figure 2: Characteristics of selected REVE-2 study patients. (A)** Patients with (LVR) or without (no LVR) left ventricle remodeling developed one year post-MI were matched for age **(**n=5/group). **(B)** Mean percentage of LVR (% LVR) at one year post-MI. % LVR was calculated as: (LVR_1year_- LVR_base_)*100/LVR_base_ **(C):** LIPCAR levels quantified by qRT-PCR in total plasma collected at one year from no LVR and LVR patients using the pair of primers LIPCAR-1. Statistical significance was determined by Wilcoxon-Mann Whitney test and only significant *p* values are indicated. **(D):** Correlation between LIPCAR levels in total plasma and % LVR. Statistical significance was determined by Spearman test. **(E):** Ejection fraction (EF) and **(F)** plasmatic levels of brain natriuretic peptide (BNP) in non LVR and LVR patients.

**Suppl. Table 1: Characteristics of selected patients from the REVE-2 study**

| **Parameters**  **( 1 year post-MI)** | **no LVR** | **LVR** |
| --- | --- | --- |
| Age | 56.4 ± 6 | 58.0 ± 6.6 |
| EF (%) | 59.2 ± 4.5 | 52.6 ± 2.2 |
| EDV mL/m2 | 45.0 ± 3.7 | 69.9 ±10.5* |
| ESV mL/m2 | 18.5 ± 2.7 | 32.9 ± 4.6* |
| % LVR | -10.1 ± 0.05 | 66.0 ± 0.1** |
| peak CK (U/L) | 1974.8 ± 450.8 | 3946.0 ± 1432 |
| BNP (pg/mL) | 30.4 ± 13.5 | 44.40 ± 16.3 |

EF: Ejection fraction, EDV: End-diastolic volume, ESV: End-systolic volume, % LVR: Percentage of left ventricle remodeling, CK: creatine kinase, BNP: brain natriuretic peptide. Statistical significance was determined by Wilcoxon-Mann Whitney test. * *P* < 0.05, ** *P* < 0.01.

**Suppl. Table 2: Number of large (lEVs) and small (sEVs) EVs isolated from HeLa conditioned culture media**

| **Series** | **lEVs (particles/cell)** | **sEVs (particles/cell)** | **ratio sEVs/lEVs** |
| --- | --- | --- | --- |
| S1 | 478.4 | 576.9 | 1.2 |
| S2 | 207.9 | 224.1 | 1.1 |
| S3 | 127.4 | 228.4 | 1.8 |
| S4 | 129.4 | 450.0 | 3.5 |

**Suppl. Table 3: LIPCAR expression in adult human cardiac tissue and human cardiac cells**

| **Human samples** | **RNA quantity for retrotranscription** | **cDNA**  **dilution** | **CT LIPCAR** | **CT GAPDH** |
| --- | --- | --- | --- | --- |
| Heart tissue | 100 ng | 1/20 | 16.3 | 19.2 |
| cardiomyocytes | 250 ng | 1/20 | 16.9 | 16.7 |
| cardiac fibroblasts | 250 ng | 1/20 | 17.5 | 17.8 |
